# Midgut bacteria in deltamethrin-resistant, deltamethrin-susceptible, and field-caught populations of *Plutella xylostella*, and phenomics of the predominant midgut bacterium *Enterococcus mundtii*

**DOI:** 10.1038/s41598-017-02138-9

**Published:** 2017-05-16

**Authors:** Wenhong Li, Daochao Jin, Caihua Shi, Fengliang Li

**Affiliations:** 1 Institute of Entomology, Guizhou University, Guiyang, 550025 P. R. China; 2 Guizhou Institute of Plant Protection, Guizhou Academy of Agricultural Sciences, Guiyang, 550006 P. R. China; 3 College of Agriculture, Yangtze University, Jingzhou, 434025 P. R. China

## Abstract

Gut bacteria play a significant role in host insect. This study evaluated detail difference of midgut bacteria in deltamethrin-resistant, deltamethrin-susceptible and field-caught populations of diamondback moth, and studied phenomics of the predominant midgut bacterium *Enterococcus mundtii*. Cultivable bacteria revealed that *E. mundtii* and *Carnobacterium maltaromaticum* dominated the bacterial populations from deltamethrin-resistant and deltamethrin-susceptible larval midguts, whereas *E*. *mundtii* was predominant in field-caught population. Illumina sequencing analysis indicated that 97% of the midgut bacteria were from the phyla Firmicutes, Proteobacteria and Cyanobacteria. Both resistant and susceptible populations had more *Enterococcus* and *Carnobacterium*. *Enterococcus, Carnobacterium*, *Bacillus*, and *Pseudomonas* were predominant in the field-caught population. A phenomics analysis revealed that *E. mundtii* was able to metabolize 25.26% of the tested carbon sources, 100% of the nitrogen sources, 100% of the phosphorus sources and 97.14% of the sulfur sources, had a wide range of osmolytes and pH conditions, and showed active deaminase activity but no decarboxylase activity. This is the first report regarding different populations of DBM midgut bacteria analyzed using both high-throughput DNA sequencing and cultivation methods, and also first report concerning the phenomics of *E. mundtii*. The phenomics of *E. mundtii* provide a basis for the future study of gut bacteria functions.

## Introduction

The diamondback moth (DBM), *Plutella xylostella* (L.) (Lepidoptera: Plutellidae), is one of the most destructive pests of cruciferous crops. It attacks economically important Cruciferae crops, including cabbage, cauliflower, broccoli, collards, mustard, rapeseed, radish and turnip^1^. The total cost connected with the damage and the management of DBM was previously estimated at 4–5 billion USD per annum worldwide^2^. Much research has been devoted to the DBM, covering pest management strategies^3,4^, the mechanism of pesticide resistance^5–7^, its developmental biology^8–10^, and herbivory mechanism^11^. However, DBM outbreaks in Southeast Asia sometimes cause more than a 90% crop loss, and this pest still seriously threatens vegetable production in China. At present, chemical control is the principal method employed to manage the DBM. Deltamethrin is one of the most commonly used pyrethroid pesticides for both agricultural and household use^12,13^. It is much effective against other insects, including *Daphnia magna*^14^, *Aedes albopictus*^15^, etc. Unfortunately, due to long-term and widespread application, deltamethrin resistance has emerged in pest insects worldwide^16,17^, especially in DBM in China^17^. Recent work has indicated that insect might have symbiont-mediated insecticide resistance. The bean bug *Riptortus pedestris* acquired bacteria of the genus *Burkholderia* from the soil, which then replace the normal *Burkholderia* midgut symbiont, conferring resistance to the insecticide fenitrothion^18^. There was a gut microbiota strongly affected the susceptibility to *Bacillus thuringiensis* (Bt) amongst lepidopteran species^19^. Other study on *Spodoptera exigua* showed that Bt resistance was associated with a higher microbiota load^20^. Though the current level of knowledge is too limited to indicate a relationship between gut symbioses and insecticide resistance, these suggest that adjusting the gut bacteria, replacing normal microbiota with exogentic one, associated with host insects may help manage their population in the future.

Insects are involved in several types of symbioses, mainly with bacteria. Bacterial association normally plays a significant role in host insect morphogenesis, food digestion, nutrition, antifungal toxin production, pheromone production, pH regulation, vitamin synthesis, temperature tolerance, resistance to parasitoid development, and the detoxification of noxious compounds^21,22^. A high diversity of bacteria has been reported in different species of insects, including *Tetanops myopaeformis*, *Lymantria dispar*, *Melanoplus sanguinipes*, *Manestra brassica* and *Helicoverpa armigera*^23–26^. Studies on the larval gut bacteria of DBM include those from Indiragandhi *et al*.^27^ and Xia *et al*.^28^. The gut bacteria *Pseudomonas* sp., *Stenotrophomonas* sp., *Acinetobacter* sp., and *Serratia marcescens* were commonly found in the prothiofos-resistant larval DBM gut; *Brachybacterium*, *Acinetobacter*, and *S. marcescens* were found in susceptible larvae; and the species *Serratia* was found in a field-caught population^27^. Additionally, *Serratia*, *Enterobacter*, *Stenotrophomonas*, and *Myroides* were also reported in the larval gut of the DBM^29^. In our laboratory, a deltamethrin-resistant DBM population and a deltamethrin-susceptible DBM population have being kept for more than 20 years^30^. However, to the best of our knowledge, no bacteria from the larval guts of deltamethrin-resistant or deltamethrin-susceptible DBM populations have been previously reported, and the detail differences of larval gut bacteria between the two laboratory populations and the field DBM population are still unknown. Moreover, the larval gut bacteria from DBM are poorly characterized and their function is still unclear. A better understanding of the metabolic characteristics of the larval gut bacteria will be very valuable in the development of management practices to decrease the impact of the DBM.

Cellular metabolic characteristics have traditionally been analyzed one at a time, and are often qualitatively and vaguely defined. Recently, a high throughput Phenotypic MicroArray/OmniLog system (PMs) was developed by Biolog (Hayward, CA, USA) to assay nearly 1000 metabolic phenotypes^31^. In the system, microorganisms are tested to analyze the use of carbon, nitrogen, sulfur and phosphorus sources, the biosynthetic pathways, and the variations of osmotic, ionic and pH. The metabolic data of microorganisms recorded by a CCD camera are quantitatively analyzed by OmniLog software. Because it could allow broad phenotypic testing at a time, provide an immediate sense of the phenotypic range of a microorganism, and is much easier to perform^31^, PM analysis has been widely used to analyze the phenotypes of many bacteria, such as *Escherichia coli*^31^, *Ralstonia solanacearum*^32^, *Bacillu subtilis*^33^, *Pseudomonas aeruginosa* and *Enterococcus faecalis*^31^.

Therefore, as an initial step towards understanding the relationship between DBM populations and their gut bacteria, this study investigated the DBM larvae midguts of (i) deltamethrin-resistant, (ii) deltamethrin-susceptible and (iii) field-caught populations to know the detail diversity of midgut bacteria in these three populations. Additionally, the dominant midgut bacterial species *E. Mundtii* isolated from the three populations was also analyzed with high throughput PMs to know its metabolic phenomics. This predominant bacterium was also found in the midgut of other insects, such as *Bombyx mori*^34^ and *Trichoplusia ni*^35^. However, the characteristics and roles of *E. mundtii* in insect are still unclear. The results would provide detail information of *E. mundtii*, and valuable knowledge about survivability of the bacterium in the gut of diamondback moth.

## Results

### Gut bacteria isolation

The highest number of bacteria per larval midgut was found in the resistant larval gut (log 6.68 CFU ml^−1^ of gut suspension) on NA medium, followed by the susceptible larval midgut (log 6.32 CFU ml^−1^ of gut suspension), the smallest in the midgut of field-caught larvae (log 5.76 CFU ml^−1^ of gut suspension). However, on LB plates, the highest number was also found in the resistant larval midgut (log 6.17 CFU ml^−1^ of gut suspension), but this was followed by the midgut of field-caught larvae (log 6.14 CFU ml^−1^ of gut suspension), and the smallest in the susceptible larval midgut (log 5.70 CFU ml^−1^ of gut suspension) (Table 1). After successive purification, a total of 18 purified gut bacterial strains were obtained, of which seven (Br-2, Br-3, Br-4, Br-5, Br-6, NBr-1 and NBr-2) were from the midgut of deltamethrin-resistant larvae, eight (M-2, M-3, M-4, M-5, M-6, NM-1, NM-2 and NM-3) from the deltamethrin-susceptible larvae, and three (T-1, NT-1 and NT-2) from the field-caught larvae (Table 2).

**Table 1:** Counts of bacteria cells hosted by the midgut of diamondback moth, *Plutella xylostella*.

Insect source	Bacterial counts in each larval midgut (log CFU ml^−1^ gut suspension)	
	Luria Bertani	Nutrient agar
Deltamethrin-resistant population	6.17 ± 0.16^b^	6.68 ± 0.26^a^
Deltamethrin-susceptible population	5.70 ± 0.10^c^	6.32 ± 0.25^a^
Field-caught population	6.14 ± 0.09^b^	5.76 ± 0.12^c^
LSD (P ≥ 0.05)	0.14	0.18

Column values followed by same letters are not significantly different from each other at 0.05%.

**Table 2:** Molecular identification of the larval gut bacteria isolated from the midgut of the diamondback moth, *Plutella xylostella*.

DBM population	Strain code	Phylogenetic neighbors			
		GenBank no. (sequence length, bp)	Close relative from GenBank (accession no.)	Media	Identity match (%)
Deltamethrin-resistant population	Br-2	KT722985	*Carnobacterium maltaromaticum*	LB	99
	Br-3	KT722986	*Carnobacterium maltaromaticum*	LB	100
	Br-4	KT722987	*Enterobacter amnigenus*	LB	99
	Br-5	KT722990	*Enterobacter amnigenus*	LB	99
	Br-6	KT722991	*Enterobacter amnigenus*	LB	100
	NBr-1	KT722996	*Enterococcus mundtii*	NA	99
	NBr-2	KT722997	*Enterococcus mundtii*	NA	99
Deltamethrin-susceptible population	M-2	KT722988	*Carnobacterium maltaromaticum*	LB	99
	M-3	KT722992	*Enterococcus mundtii*	LB	100
	M-4	KT722993	*Enterococcus mundtii*	LB	100
	M-5	KT722994	*Carnobacterium maltaromaticum*	LB	100
	M-6	KT722995	*Carnobacterium maltaromaticum*	LB	100
	NM-1	KT722998	*Enterococcus mundtii*	NA	99
	NM-2	KT722999	*Carnobacterium maltaromaticum*	NA	99
	NM-3	KT723000	*Enterococcus mundtii*	NA	100
Field-caught population	T-1	KT722989	*Enterococcus mundtii*	LB	100
	NT-1	KT723001	*Enterococcus mundtii*	NA	99
	NT-2	KT723002	*Enterococcus mundtii*	NA	99

### Molecular characterization of culturable larval midgut bacteria from the DBM

A 16S rRNA analysis revealed that the isolates obtained from the midgut of the three DBM larval were mostly belonging to three different genera (*Enterococcus*, *Enterobacter* and *Carnobacterium*) in Firmicutes and Proteobacteria (Table 2). The nucleotide sequences of the bacterial strains were subjected to homology searches in DNA databases. The results revealed that the sequences of two strains (NBr-1 and NBr-2) from the midguts of resistant larvae, four strains (M-3, M-4, NM-1, NM-3) from the midguts of susceptible larvae, and three strains (T-1, NT-1 and NT-2) from the midguts of field-caught larvae had a 99% or 100% similarity with the 16S rRNA gene sequences of *E. mundtii*. The sequences of two strains (Br-2 and Br-3) from the midguts of resistant larvae and four strains (M-2, M-5, M-6, NM-2) from the midguts of susceptible larvae had a 99% or 100% similarity with the 16S rRNA gene sequences of *C. maltaromaticum*. The sequences of three strains (Br-4, Br-5 and Br-6) from the midguts of resistant larvae showed a 99% or 100% similarity with the 16S rRNA gene sequences of *E. amnigenus*. *E. mundtii* was the predominant larval midgut bacterium obtained from the midguts of all three DBM populations.

### Sequencing results and microbial diversity in the DBM larval midgut

**Figure 1:**
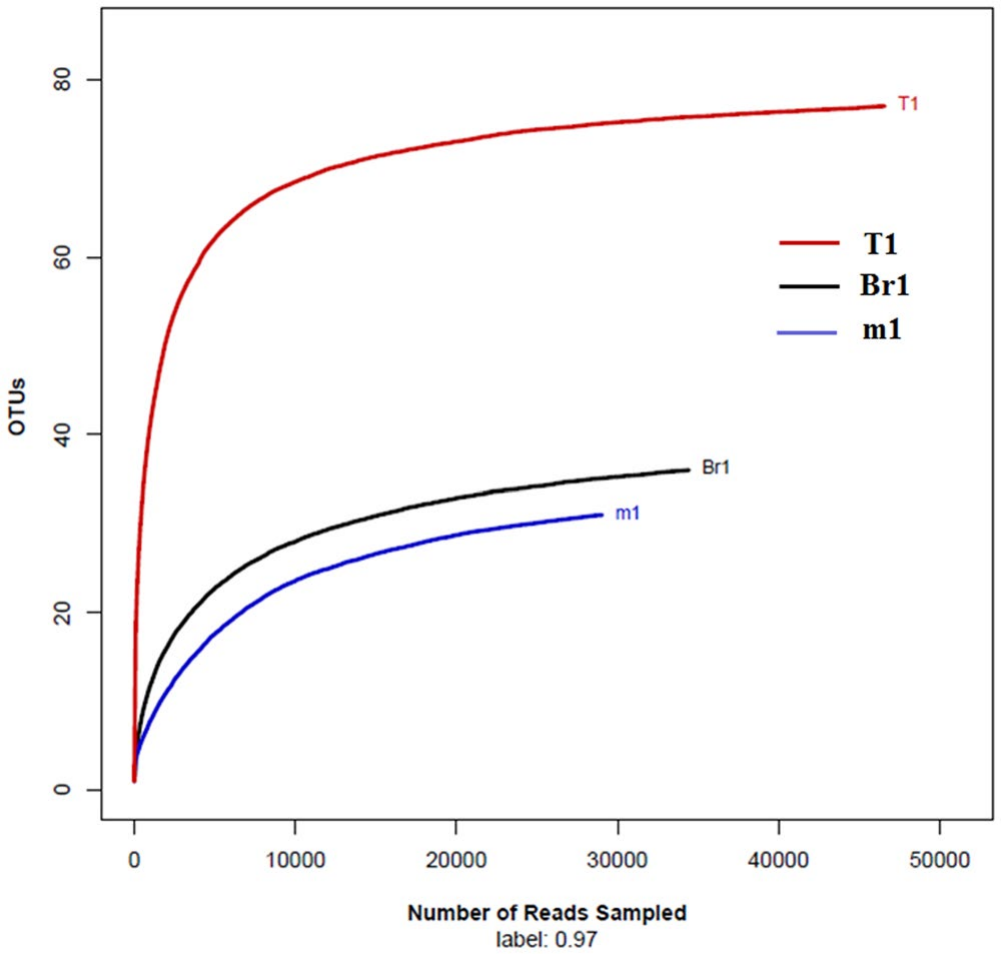
Rarefaction curves of the OTU number in a 97% similarity boxplot for each sample. T1, Br1 and m1 are shorts for the midgut samples from field-caught population, deltamethrin-resistant population and deltamethrin-susceptible population of *Plutella xylostella*, from field-caught population, deltamethrin-resistant population and deltamethrin-susceptible population, respectively.

A total of 112,321 reads and 143 OTUs were obtained from three samples through the MiSeq sequencing analysis. Each library contained 29,280 to 47,882 reads, with different phylogenetic OTUs ranging from 31 to 77. All the rarefaction curves tended to approach the saturation plateau, indicating that the data volume of the sequenced reads was reasonable, and the discovery of a high number of reads made a small contribution to the total number of OTUs. This rarefaction curve indicated the presence of a large variation in the total number of OTUs from the different samples (Fig. 1). Compared with the samples from the field-caught larvae, the samples from resistant larvae and susceptible larvae had a lower OTU density. The OTUs of sample m1 had the lowest value (31), followed by sample Br1 (35), whereas the highest was in sample T1 (77) (Table 3).

**Table 3:** MiSeq sequencing results and diversity estimates for each sample^a^.

Sample	Sequencing results		Diversity estimates		
	Total sequences	Total OTUs^a^	ACE	CHao	Shannon
m1	29280	31	33.49 ± 5.75	32.67 ± 5.87	0.69 ± 0.0106
Br1	35159	35	38.99 ± 6.66	38 ± 6.84	0.56 ± 0.0115
T1	47882	77	78.97 ± 5.09	80 ± 11.30	2.56 ± 0.0115

a: m1, Br1 and T1 are shorts for the midgut samples from deltamethrin-susceptible population, deltamethrin-resistant population and field-caught population of *Plutella xylostella*, respectively.

The alpha diversity species richness (Chao), evenness (ACE), and Shannon index all confirmed the highest diversity in sample T1 from the field-caught larvae and less diversity in the samples from resistant (Br1) and susceptible larvae (m1) (Table 3). The Shannon diversity indices of the three samples were 2.56 (T1), 0.69 (m1), and 0.56 (Br1), indicating that the Shannon diversity of the sample from field-caught larvae was significantly higher than that of the other two samples.

### Taxonomic composition of the samples from the DBM larval midgut

**Figure 2:**
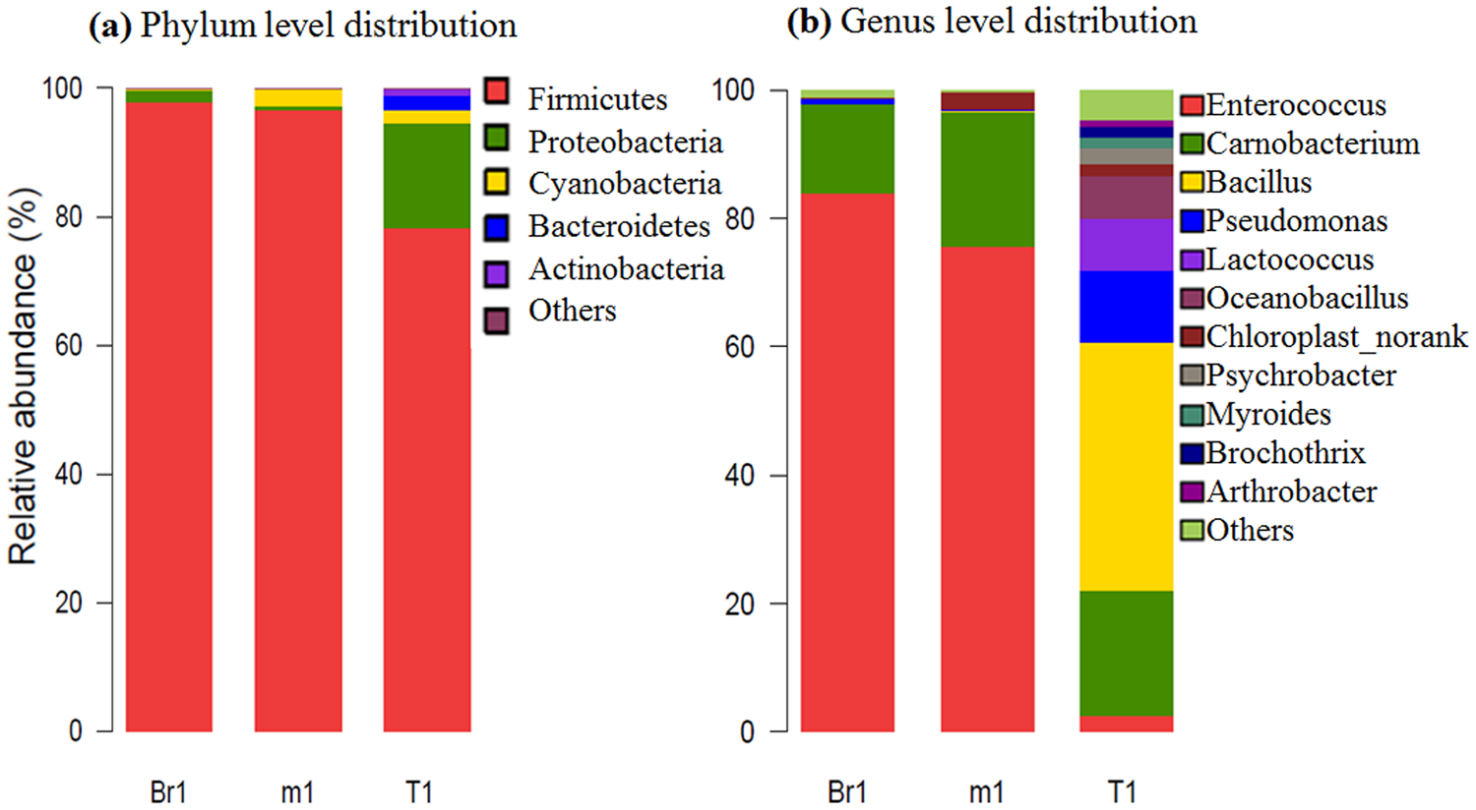
Taxonomic distribution of larval midgut samples. (**a**) Phylum distribution for all samples; (**b**) genus distribution of all samples.

Sequences that could not be classified into any known group were unclassified. The bacterial OTUs were assigned to 11 genera, 5 different phyla. Two phyla (Firmicutes and Proteobacteria) out of 5 total phylotypes were common to the three samples, which comprised more than 95% of the total reads in every library. Firmicutes was the most abundant group (Fig. 2a), comprising approximately 22.22% (32) of the OTUs and 89.21% (98,197) of the reads across all samples. Proteobacteria, the second most abundant phylum, (59.72%, 86 OTUs) comprised 7.70% (8,479 reads) in all libraries. However, the OTU proportion of Firmicutes in the different samples showed high variability, ranging from 11.11% to 29.87%. The read proportions of Firmicutes in samples T1, m1 and Br1 were 78.22%, 96.68% and 97.77%, respectively, whereas the read proportions of Proteobacteria were 16.44%, 0.58% and 1.89%, respectively (Fig. 2a). Additionally, Bacteroidetes was detected only in a sample (T1) from field-caught larvae and not in the other two samples. Cyanobacteria (4.17%, 6 OTUs), Bacteroidetes (7.64%, 11 OTUs), and Actinobacteria (3.47%, 7 OTUs) comprised 1.62% (1,787 reads), 0.95% (1,046 reads) and 0.48% (533 reads) in all libraries, respectively.

Two genera (*Enterococcus* and *Carnobacterium*) of the eleven detected genera were common to all three samples, comprising more than 95% of the total reads in the libraries from samples Br1 and m1 and approximately 20% of the total reads in the library from sample T1. For sample m1 from susceptible larvae and sample Br1 from resistant larvae, *Enterococcus* was the most abundant group (Fig. 2b), comprising approximately 3.01% (2) of the OTUs and 80.15% (50,878) of the reads across all samples; *Carnobacterium*, the second most abundant genus (3.01%, 2 OTUs), comprised 17.11% (10,863 reads) in all libraries. The read proportions of *Enterococcus* in samples T1, m1 and Br1 were 2.55%, 75.60% and 83.99%, respectively, whereas the read proportions of *Carnobacterium* were 19.62%, 21.07% and 13.77%, respectively (Fig. 2b). Additionally, the read proportions of *Bacillus*, *Pseudomonas*, *Lactococcus*, *Oceanobacillus*, *Chloroplast_norank*, *Psychrobacter*, *Myroides*, *Brochothrix* and *Arthrobacter* in sample T1 were 5.19%, 3.90%, 2.60%, 1.20%, 2.60%, 2.60%, 2.60%, 1.30% and 1.30%, respectively, whereas much lower read proportions of these genera were detected in samples m1 and Br1.

### Differences between the bacterial genera in different midgut samples

**Figure 3:**
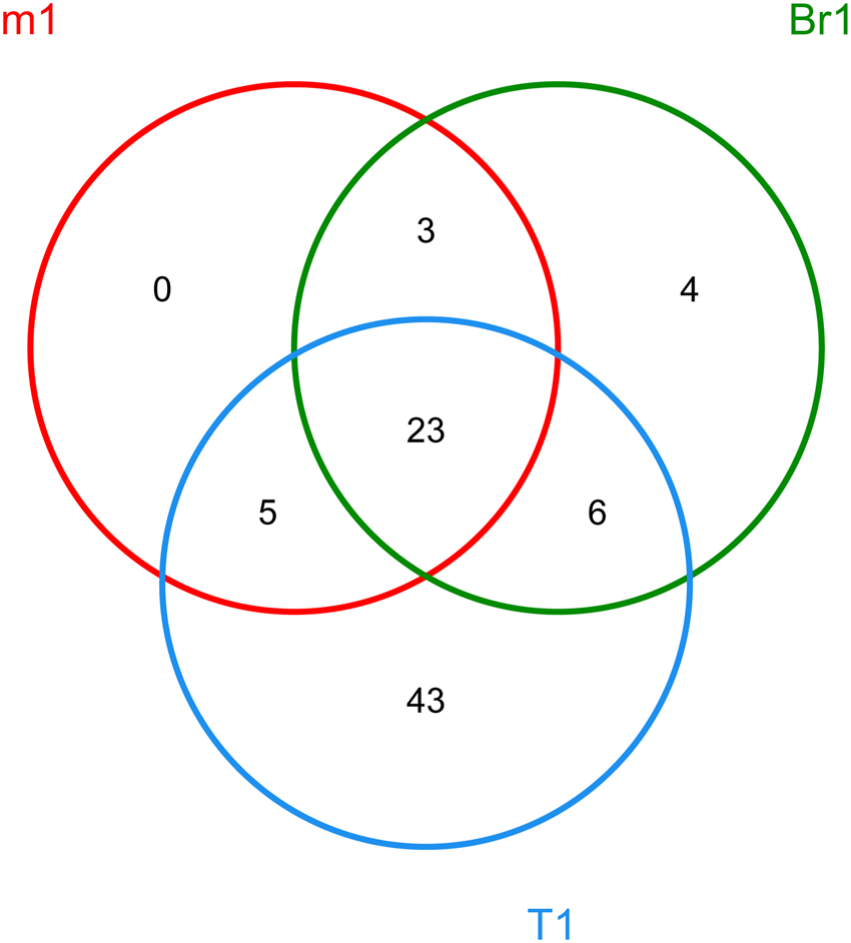
Venn diagram showing the bacterial genera detected in the three different samples m1, Br1, and T1. Overlaps between the samples are indicated by the arrangement of the circles.

At the genus level, the differences between the communities from different gut samples were depicted with a Venn diagram. A total of 84 genera were discovered, and 27.38% of them were shared genera (Fig. 3). Sample T1 contained more bacterial varieties (77 genera) than samples Br1 (36 genera) and m1 (31 genera), as shown in Fig. 3. An overlap between the genera detected in the three samples was also observed. The largest overlap was found between samples Br1/T1 (29 genera), followed by samples m1/T1 (28 genera) and samples m1/Br1 (26 genera).

**Figure 4:**
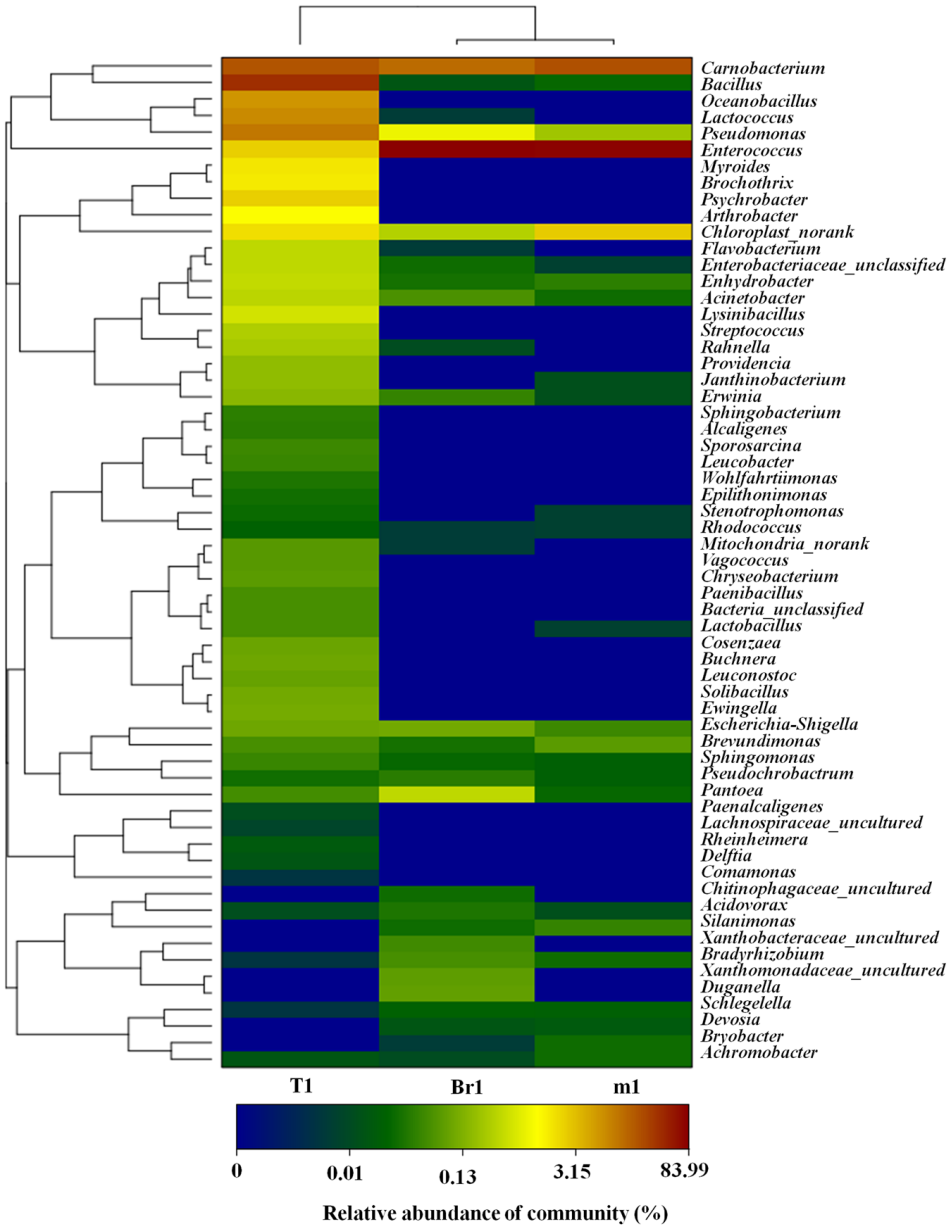
Bacterial distribution of the abundant genera in three samples. The bacterial phylogenetic tree was calculated using the neighbor-joining method. The heatmap plot depicts the relative abundance of each bacterial genus (variables clustering on the vertical axis) within each sample. The relative values for the bacterial genera are indicated by the color intensity with the legend in the top right corner.

Based on the relative abundance of the genera as shown in Fig. 4, genera with an average abundance of >1% in at least one sample were defined as predominant. The Venn diagram indicates that four dominant genera belonged to the genera shared by three samples (m1, Br1 and T1), including *Carnobacterium*, *Pseudomonas*, *Enterococcus* and *Chloroplast_norank*. In sample Br1, the genus *Pantoea* was also predominant, whereas for sample T1, seven other predominant genera were found, including *Bacillus*, *Oceanobacillus*, *Lactococcus*, *Myroldes*, *Brochothrix*, *Psychrobacter* and *Arthrobacter*. The relative abundances of the genera *Bacillus*, *Pseudomonas*, *Lactococcus*, *Oceanobacillus* and *Psychrobacter* in sample T1 were much higher than in samples m1 and Br1. Additionally, the relative abundance of the genus *Enterococcus* in samples m1 and Br1 was greater than 3% and significantly higher than in sample T1.

### Phenotypic characterization

**Figure 5:**
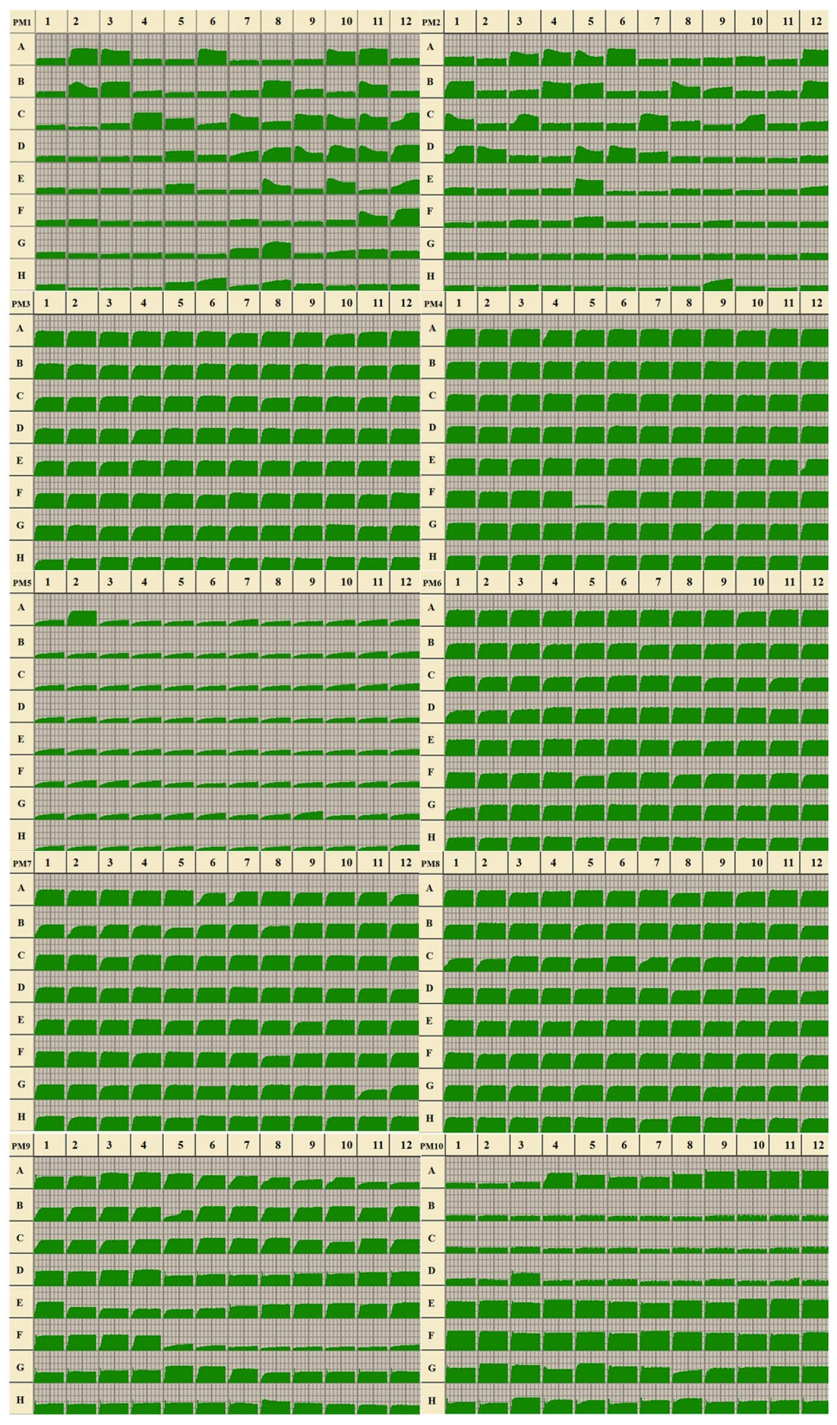
Data for Biolog Phenotype MicroArray PM 1–10 plates of the gut bacteria *Enterococcus mundtii* NT-1. (Utilization of the isolate of *E. mundtii* from the DBM gut is indicated by green areas in the growth curve for each substrate. The threshold to consider effective growth of *E. mundtii* was quantitatively analyzed by Biolog OmniLog software that tested the color value of each well).

Isolate NT-1 of *E. mundtii* had a typical phenotypic fingerprint. This species was able to metabolize 25.26% of the carbon sources tested (29/95 in plate PM1 and 19/95 in plate PM2), 100% of the nitrogen sources (95/95 in plate PM3, 95/95 in plate PM6, 95/95 in plate PM7, and 95/95 in plate PM8), 100% of the phosphorus sources (59/59 in plate PM4, Wells A02-E12), and 97.14% of the sulfur sources (34/35 in plate PM4, Wells F02-H12) (Fig. 5). The sole sulfur source that could not be metabolized by *E. mundtii* was thiophosphate (PM4, Well F05). Additionally, the species contained none of the biosynthetic pathways tested (0/94 in plate PM5).

The data from PM1 and PM2 (carbon sources) indicate that *E. mundtii* was able to use 48 different carbon sources (Fig. 5, Table 4). In between, around forty compounds were effectively utilized by *E. mundtii*, including L-arabinose, N-acetyl-D-glucosamine, D-galactose, D-trehalose, D-mannose, D-sorbitol, D-xylose, D-mannitol, D-ribose, D-fructose, α-D-glucose, etc. (Table 4). In comparison, approximately 140 compounds could not be utilized by this gut bacterial species (Fig. 5).

**Table 4:** Carbon substrates in the PM 1–2 Biolog MicroPlates metabolized by the larval gut bacterium *Enterococcus mundtii* from the diamondback moth^a^.

Well	Substrate	Well	Substrate	Well	Substrate
PM1					
A02	L-Arabinose	C07	D-Fructose	E05	Tween 80
A03	N-Acetyl-D-Glucosamine	C09	α-D-Glucose	E08	ß-Methyl-D-Glucoside
A06	D-Galactose	C10	Maltose	E10	Maltotriose
A10	D-Trehalose	C11	D-Melibiose	E12	Adenosine
A11	D-Mannose	C12	Thymidine	F11	D-Cellobiose
B02	D-Sorbitol	D08	α-Methyl-D-Galactoside	F12	Inosine
B03	Glycerol	D09	α-D-Lactose	G07	Acetoacetic acid
B08	D-Xylose	D10	Lactulose	G08	N-Acetyl-D-Mannosamine
B11	D-Mannitol	D11	Sucrose	H06	L-Lyxose
C04	D-Ribose	D12	Uridine		
PM2					
A03	α-Cyclodextrin	B08	Arbutin	D02	Salicin
A04	ß-Cyclodextrin	B12	3–0-ß-D-Galactopyranosyl-D-Arabinose	D05	Stachyose
A05	γ-Cyclodextrin	C01	Gentiobiose	D06	D-Tagatose
A12	Pectin	C03	D-Lactitol	E05	D-Glucosamine
B01	N-Acetyl-D-Galactosamine	C07	ß-Methyl-D-Galactoside	H09	Dihydroxyacetone
B04	Amygdalin	C10	α-Methyl-D-Mannoside		
B05	D-Arabinose	D01	D-Raffinose		

^a^Content listed in the columns “Well” and “Substrate” refer to the layout of the Biolog PM Microplate; the number in the Well column indicate the substrates tested in the Biolog PM Microplate.

The PM3 plate tested isolate NT-1 of *E. mundtii* for its ability to grow on 95 different nitrogen sources (amino acids) (Fig. 5). All these compounds were utilized by *E. mundtii*, including L-cysteine, uric acid, etc. (Fig. 5). Meanwhile the PM6, PM7 and PM8 plates tested *E. mundtii* for its ability to grow on 285 different nitrogen pathways. All the tested compounds could also be utilized by *E. mundtii* (Fig. 5).

Plates PM9 and PM10 were used to test for growth under various stress conditions. *E. mundtii* showed active metabolism with up to 8% sodium chloride, 6% potassium chloride, 5% sodium sulfate, 20% ethylene glycol, 6% sodium formate, 7% urea, 4% sodium lactate, 200 mM sodium phosphate (pH 7.0), 200 mM sodium benzoate (pH 5.2), 100 mM ammonium sulfate (pH 8.0), 100 mM sodium nitrate, and 100 mM sodium nitrite, but it could not metabolize sodium lactate, which ranged from 5% to 12% (plate PM9, Well F05 to F12) in our analysis (Fig. 5, Table 5). When combined with various osmolytes in 6% sodium chloride, *E. mundtii* grew well in all tests (plate PM9, Well B01 to B12, and C01 to C12). The pH range for the active growth of *E. mundtii* was between 5 and 10, with an optimal pH of approximately 10.0. When combined with various amino acids at a pH of 4.5, *E. mundtii* showed no growth in any test except when combined with the amino acid L-norvaline (plate PM10, well D03) (Fig. 5, PM10). In comparison, when combined with various amino acids at a pH of 9.5, *E. mundtii* grew actively in all tests. PM10, wells B1-D12 and E1-G12, tested the decarboxylase and deaminase activities of *E. mundtii* in the presence of amino acids at pH 4.5 and pH 9.5, respectively (Table 6). In the presence of most the amino acids, *E. mundtii* showed active deaminase activity but no decarboxylase activity (Fig. 5, PM10).

**Table 5:** Substrates in the PM 9 Biolog MicroPlate metabolized by the larval gut bacterium *Enterococcus mundtii* from the diamondback moth^a^.

Well	Substrate	Well	Substrate	Well	Substrate	Well	Substrate
A01	1% NaCl	C01	6% NaCl + KCl	D11	15% Ethylene glycol	G05	50 mM Sodium benzoate pH 5.2
A02	2% NaCl	C02	6% NaCl + L-proline	D12	20% Ethylene glycol	G06	50 mM Sodium benzoate pH 5.2
A03	3% NaCl	C03	6% NaCl + N-acetyl L-glutamine	E01	1% Sodium formate	G07	100 mM Sodium benzoate pH 5.2
A04	4% NaCl	C04	6% NaCl + β-glutamic acid	E02	2% Sodium formate	G08	200 mM Sodium benzoate pH 5.2
A05	5% NaCl	C05	6% NaCl + γ-amino-n-butyric acid	E03	3% Sodium formate	G09	10 mM Ammonium sulfate pH 8
A06	5.5% NaCl	C06	6% NaCl + glutathione	E04	4% Sodium formate	G10	20 mM Ammonium sulfate pH 8
A07	6% NaCl	C07	6% NaCl + glycerol	E05	5% Sodium formate	G11	50 mM Ammonium sulfate pH 8
A08	6.5% NaCl	C08	6% NaCl + trehalose	E06	6% Sodium formate	G12	100 mM Ammonium sulfate pH 8
A09	7% NaCl	C09	6% NaCl + trimethylamine-N-oxide	E07	2% Urea	H01	10 mM Sodium nitrate
A10	8% NaCl	C10	6% NaCl + trimethylamine	E08	3% Urea	H02	20 mM Sodium nitrate
B01	6% NaCl	C11	6% NaCl + octopine	E09	4% Urea	H03	40 mM Sodium nitrate
B02	6% NaCl + betaine	C12	6% NaCl + trigonelline	E10	5% Urea	H04	60 mM Sodium nitrate
B03	6% NaCl + N-N dimethyl glycine	D01	3% Potassium chloride	E11	6% Urea	H05	80 mM Sodium nitrate
B04	6% NaCl + sarcosine	D02	4% Potassium chloride	E12	7% Urea	H06	100 mM Sodium nitrate
B05	6% NaCl + dimethyl sulphonyl propionate	D03	5% Potassium chloride	F01	1% Sodium lactate	H07	10 mM Sodium nitrite
B06	6% NaCl + MOPS	D04	6% Potassium chloride	F02	2% Sodium lactate	H08	20 mM Sodium nitrite
B07	6% NaCl + ectoine	D05	2% Sodium sulfate	F03	3% Sodium lactate	H09	40 mM Sodium nitrite
B08	6% NaCl + choline	D06	3% Sodium sulfate	F04	4% Sodium lactate	H10	60 mM Sodium nitrite
B09	6% NaCl + phosphoryl choline	D07	4% Sodium sulfate	G01	20 mM Sodium phosphate pH 7	H11	80 mM Sodium nitrite
B10	6% NaCl + creatine	D08	5% Sodium sulfate	G02	50 mM Sodium phosphate pH 7	H12	100 mM Sodium nitrite
B11	6% NaCl + creatinine	D09	5% Ethylene glycol	G03	100 mM Sodium phosphate pH 7		
B12	6% NaCl + L-carnitine	D10	10% Ethylene glycol	G04	200 mM Sodium phosphate pH 7		

^a^Content listed in the columns “Well” and “Substrate” refer to the layout of the Biolog PM Microplate; the numbers in the Well column indicate the substrates tested in the Biolog PM Microplate.

**Table 6:** Substrates in the PM 10 Biolog MicroPlate metabolized by the larval gut bacterium *Enterococcus mundtii* from the diamondback moth^a^.

Well	Substrate	Well	Substrate	Well	Substrate
A04	pH 5	E11	pH 9.5 + L-leucine	G07	pH 9.5 + histamine
A05	pH 5.5	E12	pH 9.5 + L-lysine	G08	pH 9.5 + phenylethylamine
A06	pH 6	F01	pH 9.5 + L-methionine	G09	pH 9.5 + tyramine
A07	pH 7	F02	pH 9.5 + L-phenylalanine	G10	pH 9.5 + tryptamine
A08	pH 8	F03	pH 9.5 + L-proline	G11	pH 9.5 + trimethylamine-N-oxide
A09	pH 8.5	F04	pH 9.5 + L-serine	G12	pH 9.5 + urea
A10	pH 9	F05	pH 9.5 + L-threonine	H01	X-caprylate
A11	pH 9.5	F06	pH 9.5 + L-tryptophan	H02	X-α-D-glucoside
A12	pH 10	F07	pH 9.5 + L-tyrosine	H03	X-ß-D-glucoside
D03	pH 4.5 + α-amino-N-butyric acid	F08	pH 9.5 + L-valine	H04	X-α-D-galactoside
E01	pH 9.5	F09	pH 9.5 + hydroxy-L-proline	H05	X-ß-D-galactoside
E02	pH 9.5 + L-alanine	F10	pH 9.5 + L-ornithine	H06	X-α-D-glucuronide
E03	pH 9.5 + L-arginine	F11	pH 9.5 + L-homoarginine	H07	X-ß-D-glucuronide
E04	pH 9.5 + L-asparagine	F12	pH 9.5 + L-homoserine	H08	X-ß-D-glucosaminide
E05	pH 9.5 + L-aspartic acid	G01	pH 9.5 + anthranilic acid	H09	X-ß-D-galactosaminide
E06	pH 9.5 + L-glutamic acid	G02	pH 9.5 + L-norleucine	H10	X-α-D-mannoside
E07	pH 9.5 + L-glutamine	G03	pH 9.5 + L-norvaline	H11	X-PO4
E08	pH 9.5 + glycine	G04	pH 9.5 + agmatine	H12	X-SO4
E09	pH 9.5 + L-histidine	G05	pH 9.5 + cadaverine		
E10	pH 9.5 + L-isoleucine	G06	pH 9.5 + putrescine		

^a^Content listed in the columns “Well” and “Substrate” refer to the layout of the Biolog PM Microplate; the numbers in the Well column indicate the substrates tested in the Biolog PM Microplate.

## Discussion

To understand the contribution of gut bacteria to host processes, it is necessary to determine the bacterial structure and diversity and the metabolic phenotypic characterization of some of the predominant bacteria in the host insect gut environment. This study investigated the larval midgut bacteria from deltamethrin-resistant, deltamethrin-susceptible and field-caught DBM populations and also characterized the metabolic phenotypes of the dominant midgut bacterial species *E. mundtii*.

A statistical analysis of the cultivable bacterial populations obtained on two different media did not show significant differences in the bacterial populations, indicating that the media composition did not appear to affect the cultivable bacterial strains, as previously demonstrated^27,36^. Only three bacterial genera were obtained in the present study, indicating that cultivation-dependent methods have limitations for bacterial diversity studies and do not reflect the actual quantitative relationships in the DBM larval midgut. Similar findings have also been previously reported^27,29^. Meanwhile, the bacterial genera obtained by the cultivation-dependent method used in this study have also been documented as present in the gut of the DBM^27,29,37^ and other Lepidopteran families as well^25,34,35,38^. The field-caught population of DBM larvae harbored single phylotypes of *E. mundtii*, and similar bacterial monoassociations have been reported in some other insect orders, including Orthoptera, Thysanoptera, and Hemiptera^36,39,40^. A monoassociation of predominant bacteria might eliminate or prevent the colonization of other competitive micro-organisms.

The sequencing results in this study showed that DBM midgut bacteria were diverse, but only two microbial phyla (Firmicutes and Proteobacteria) were predominant, as were three genera (*Enterococcus*, *Carnobacterium*, and *Bacillus*). Similar results have also been found in eight species of mosquitoes^41^ and the midguts of other Lepidoptera, including *Lymantria dispar*, *Helicoverpa armigera*, and *Bombyx mori*^25,26,38^. The most abundant genera in the DBM larval midgut were *Enterococcus* and *Carnobacterium*. It has been reported that the capacity of these genera to degrade carbohydrates could be useful to the digestion of the host insect^42^, and this function should be tested in the DBM.

Larval midgut bacteria of the deltamethrin-resistant, deltamethrin-susceptible and field-caught DBM populations had different structures and diversity patterns according to Illumina sequencing in this study. They should have had a similar structure prior to insecticide exposure. The midgut bacteria from field-caught populations were more diverse, and this structure should be much nearer to the actual midgut bacterial structure of the DBM in the field in Guizhou province in China. The structure and diversity of midgut bacteria from the deltamethrin-resistant and deltamethrin-susceptible populations were similar in some cases, possibly due to nearly 20 years in a similar rearing environment in our laboratory^30^; however, the differences at the genera level were large. Although these could be a consequence of exposure to insecticides that had differential toxicities to different bacterial taxa, it is important to consider the possibility of the role of certain larval midgut bacteria related to deltamethrin resistance. The susceptible population exhibited a higher proportion of bacteria from the phylum Cyanobacteria than the resistant population. The resistant population had a higher proportion of bacteria from the genus *Pseudomonas* than the susceptible population. Additionally, some of the less abundant bacteria varied markedly between the susceptible and resistant populations. These differences might be due to the chemical environment in the gut. Similar findings have been reported by others. Symbiont-mediated insecticide resistance has been demonstrated in stinkbugs^18^. Symbiotic *Burkholderia* from the soil have been shown to enhance the resistance of *Riptortus pedestris*^43^. The differences in bacterial taxa in the larval midgut from three different DBM populations in this study indicate that further work to identify the possible reasons for deltamethrin resistance in the DBM is warranted.

Although a few studies have been performed on the gut bacteria of Lepidoptera, they have dealt with only the isolation and characterization of the microbial flora^26,27,29^. However, this study involved the characterization of the metabolic phenotype of the predominant midgut bacterium *E. mundtii*, revealing significant metabolic diversity. Many carbon compounds could be utilized, and most nitrogen, sulfur, and phosphorus sources were also metabolized. These data indicate the great versatility of *E. mundtii* in the DBM gut environment. The most informative plates for *E. mundtii* were PM1/PM2 (carbon sources), PM9 (osmolyte conditions) and PM10 (pH conditions). The most informative utilization patterns for carbon sources were saccharides and for nitrogen sources were various amino acids and peptides. These compounds are commonly found in many plant leaves. They might play a key role in the survival of *E. mundtii* and thus in supporting the digestion of food in the DBM. Additionally, *E. mundtii* had wide range of tolerance to various osmolytes and pH conditions, as indicated by plates PM9 and PM10. Bacterial deaminases generate acid via the catabolism of amino acids, which help counteract an alkaline pH^44,45^. The phenotypic diversity of *E. mundtii* can be explained by considering the seasonal variation in osmolytes and gut pH due to dietary variation of the DBM. Consequently, phenotypic characteristics for the utilization of those sources and the wide range of tolerances of *E. mundtii* could have a high adaptive value in host-microbe interactions and the survival of the bacterium in the DBM gut.

In conclusion, although there is insufficient evidence to demonstrate whether certain bacterial taxa are responsible for conferring DBM deltamethrin resistance and whether such a mechanism works together with other mechanisms (insect physiology changes or gene mutation), the data obtained in this study still provide useful information about the molecular characterization of insect midgut bacteria and its relationship with the important phenomenon of insecticide resistance. The phenotypic characterization of the dominant midgut bacterium *E. mundtii* could also help us understand its potential role in the DBM midgut. Given the significant damage that the DBM causes worldwide and the difficulty in controlling it due to insecticide resistance, the roles of some predominant bacteria and the possibility that microbial symbiont-mediated resistance is active in this insect should be further investigated.

## Materials and Methods

### Collection and mass rearing of insects

Deltamethrin-resistant and -susceptible populations of DBM larva (Br1, m1), able to tolerate >1000 μg ml^−1^ and 3 μg ml^−1^ of deltamethrin, respectively, were obtained in a previous study^30^. The field-caught population of DBM larva (T1) was collected from a cabbage field at the Guizhou Academy of Agricultural Sciences in Guizhou province in China, where no insecticides had been applied to control the DBM. This population was also able to tolerate 3 μg ml^−1^ of deltamethrin, and was chosen as a control population when compared with the laboratory populations. Larvae from the three populations were reared in a sterile acryl cages (45 × 45 × 50 cm) with cabbage (*Brassica oleracea* L.) and maintained at 18–25 °C and a 50–60% relative humidity under 16 h of light and 8 h of darkness. The cabbage leaves were washed with 70% ethanol for 60 s followed by 5% NaOCl (60 s), thoroughly rinsed with distilled water to remove the disinfectant, then air dried and used to rear the insects.

### Midgut sampling and the isolation of cultivable bacteria

The most destructive third instars of the four larval stages of the DBM were selected for the isolation of midgut bacteria^27,46^. A total of 40 third instars from each population were selected and starved for 24 h. The starved larvae were surface disinfected with 70% ethanol for 60 s followed by 5% NaOCl for 60 s, thoroughly rinsed with distilled water to remove the disinfectant, and the midgut contents of the larvae were isolated and were homogenized with 2 ml of 0.1 M phosphate buffer (pH 7.0).

Portions (0.1 ml of each midgut suspension (diluted 10^−1^) were transferred to 4.5 ml of sterile distilled water and subsequently diluted to 10^−2^, 10^−3^, 10^−4^ and 10^−5^. One hundred microliters of aliquots of the 10^−3^ to the 10^−6^ midgut dilutions were inoculated onto the surface of Luria Bertani (LB) and nutrient agar (NA) plates^27,36^. After inoculation, the Petri dishes were placed at 30 °C in the dark for 48 h. The number of colonies formed on each Petri dish was counted. The number of cultivable bacteria for each DBM midgut population was calculated. Bacteria of different colors, growth rates and morphologies were selected from the agar plates, and a single representative isolate of each morphotype was transferred to a new plate. After five to six successive passages, the purified strains were maintained in 30% glycerol at −20 °C for long term storage. The bacteria were revived on LB agar before use in a study.

### Molecular identification of culturable midgut bacteria

A loopful of each midgut bacterial colony from an LB agar plate was picked, re-suspended in 150 µl of distilled water, boiled for 12 min in an Eppendorf tube, cooled to room temperature on ice for 8 min, centrifuged at 9000 g for 2 min and the supernatant was utilized for PCR. The 16S rRNA gene of each bacterium was amplified via PCR using the forward primer 27F (5′-AGAGTTTGATCCTGGCTCAG-3′) and reverse primer 1492R (5′-GGTTACCTTGTTACGACTT-3′). The PCR amplifications were conducted in a thermocycler (Biorad MyCycler; BioRad, CA, USA) in a 30-µl reaction system that contained 6 µl of boiled supernatant, 3 mM MgCl_2_, 0.5 µM of each primer, 1 unit of *Taq* DNA polymerase (Takara, Dalian, China), and 200 µM (each) deoxynucleoside triphosphate (dNTP) in 1× PCR buffer. PCR conditions were as follows: 95 °C for 10 min, 35 cycles of 30 s at 95 °C, 120 s at 63 °C, and 1 min at 72 °C, and a final extension at 72 °C for 10 min. Products (4 µl) were loaded on 1.0% agarose (Biowest, Spain) gels, electrophoresed at 100 V for 30 min and checked under UV transillumination (254 nm). The 16S rRNA nucleotide sequences were sequenced by Shanghai Sangon Biotech Company. The nucleotide sequences obtained were submitted to the NCBI database and their accession numbers (KT722985 to KT723002) are available in GenBank.

### Collection of larval midgut contents

To collect the midgut contents, 40 third instars larvae were randomly sampled from each insect line, regardless of sex. The larvae were surface-sterilized with 75% ethanol for 90 sec and rinsed with sterile, deionized water. After dissection, the midgut contents were homogenized with 1 ml of distilled water and frozen at −80 °C before DNA extraction.

### DNA extraction and PCR amplification of the V3-V4 region of 16S rRNA

Total bacterial DNA from the DBM larval midgut was extracted using the PowerSoil DNA Isolation Kit (MO BIO laboratories, San Diego, USA) following the manufacturer’s instructions with some modifications. The midgut contents were placed into liquid nitrogen and thawed at 37 °C before cell lysis. After adding the C1 solution (a component of the kit), the sample was completely homogenized by vortexing for 20 min. Other subsequent steps were conducted following the manufacturer’s protocol. The DNA products were checked on 1.0% agarose gels. A region of approximately 460 bp in the 16S rRNA gene and covering the V3-V4 regions was selected to construct a community library. The broadly conserved primers 338F (5′- ACTCCTACGGGAGGCAGCA-3′) and 806R (5′-GGACTACHVGGGTWTCTAAT-3′) were used to amplify this region^47^. PCR was carried out in a total volume of 20 μl: 11.5 μl of H_2_O, 4 μl of 5 × FastPfu Buffer, 0.5 μl of DNA template (100 ng/μl), 2 μl of 2.5 mM dNTPs, 0.8 μl of 338 F (5 μM), 0.8 μl of 806 R (5 μM), and 0.4 μl of FastPfu Polymerase. After initial denaturation at 95 °C for 3 min, amplification was performed using 27 cycles of 30 sec at 95 °C, 30 sec at 55 °C, and 45 sec at 72 °C, followed by a final extension at 72 °C for 10 min. Negative controls were conducted as described above but without the DNA template. The amplification products were then run on 1.0% agarose gels and purified, and the products were sent to Majorbio in Shanghai for construction of the V3-V4 library for sequencing.

### Illumina MiSeq sequencing of the 16S rRNA V3-V4 region and data analysis

The PCR products were purified using the AxyPrep DNA Gel Extraction Kit (Axygen Biosciences, Union City, CA, USA), end-repaired, A-tailed, PE-adapter ligated and then sequenced on an Illumina MiSeq PE300. Clean data were generated after trimming and removing reads with low quality scores, then PE reads were overlapped to full V3-V4 tags. Tags with lengths of less than 50 bp were removed for further analysis. The redundant tags were deleted by Mothur v. 1.30.1^48^, and unique tags were obtained. The unique tags were aligned against the 16S rRNA V3-V4 database^49^ using the BLASTN algorithm. Operational taxonomic units (OTUs) with a 97% similarity cutoff were clustered using UPARSE (version 7.1), and chimeric sequences were identified and removed using UCHIME.

Rarefaction analysis based on Mothur v. 1.30.1^48^ was conducted to reveal the diversity indices, including the Chao, ACE, and Shannon diversity indices. Venn diagrams were implemented by Venn Diagram, whereas a Mantel test, a redundancy analysis (RDA), and Heatmap figures were performed in the Vegan package in R.

### Phenotypic characterization

The primary gut bacterium *E. mundtii* isolated from all three DBM populations was analyzed with high throughput PMs (Biolog, Hayward, CA, USA) according to a published procedure^31,50,51^. The isolate NT-1 was chosen randomly from the *E. mundtii* isolates and analyzed in this study. All materials, reagents and media for the phenotypic study were purchased from Biolog. The isolate was streaked on Biolog Universal Growth medium plus blood agar (BUG + B) plates and incubated at 30 °C in the dark for 48 h. The cells were scraped from the surface of the plates and re-suspended in an appropriate medium containing Dye Mix F; 100 µl of a 1:200 dilution of a cell suspension at 81% transmittance was added to each well of the PM plates. Plates 1–8, which tested for the phenotypes of carbon, nitrogen, phosphorus, and sulfur utilization, as well as for biosynthetic pathways, and plates 9–10, which tested for osmotic/ion and pH effects, were used in this study. IF-0a GN/GP Base inoculating fluid was prepared for PM plates 1–8. IF-10b GN/GP Base inoculating fluid was utilized for plates 9 and 10. After inoculation in a laminar flow hood, the plates were incubated in the OmniLog incubator for 72 h. Data were collected every 15 min by the Biolog incubator and analyzed using the Biolog Kinetic and Parametric software. Phenotype diversities were evaluated based on the area differences under the kinetic curves of color formation. The experiment was conducted twice.

### Statistical analysis

All statistical analyses were conducted using SPSS 14.0 (SPSS Inc., Chicago). A mean comparison was conducted using the least significant difference (LSD) *P* ≤ 0.05.
