# Author Correction: Midgut bacteria in deltamethrin-resistant, deltamethrin-susceptible, and field-caught populations of *Plutella xylostella*, and phenomics of the predominant midgut bacterium *Enterococcus mundtii*

**DOI:** 10.1038/s41598-018-23329-y

**Published:** 2018-03-19

**Authors:** Wenhong Li, Daochao Jin, Caihua Shi, Fengliang Li

**Affiliations:** 1 Institute of Entomology, Guizhou University, Guiyang, 550025 P. R. China; 2 Guizhou Institute of Plant Protection, Guizhou Academy of Agricultural Sciences, Guiyang, 550006 P. R. China; 3 College of Agriculture, Yangtze University, Jingzhou, 434025 P. R. China

Correction to: *Scientific Reports* 10.1038/s41598-017-02138-9, published online 16 May 2017

In the original version of this Article, Affiliation 2 was incomplete. The correct Affiliation is listed below:

Guizhou Institute of Plant Protection, Guizhou Academy of Agricultural Sciences, Guiyang, 550006, P.R. China.

This error has now been corrected in the PDF and HTML versions of the Article.

